# Non-Steroidal Anti-Inflammatory Drugs and Brain Inflammation: Effects on Microglial Functions

**DOI:** 10.3390/ph3061949

**Published:** 2010-06-14

**Authors:** Maria Antonietta Ajmone-Cat, Antonietta Bernardo, Anita Greco, Luisa Minghetti

**Affiliations:** Experimental Neurology Section, Department of Cell Biology and Neurosciences, Istituto Superiore di Sanità, Viale Regina Elena 299, 00161 Rome, Italy; E-Mails: mariaantonietta.ajmone-cat@iss.it (M.A.A-C.); antonietta.bernardo@iss.it (A.B.); anita.greco@iss.it (A.G.)

**Keywords:** brain, cyclooxygenase, microglia, neuroprotection, NSAIDs, PPAR-γ, transcription factors

## Abstract

The term NSAID refers to structurally diverse chemical compounds that share the ability to inhibit the activity of the prostaglandin (PG) biosynthetic enzymes, the cyclooxygenase (COX) isoforms 1 and 2. The suppression of PG synthesis at sites of inflammation has been regarded as primarily responsible for the beneficial properties of NSAIDs, but several COX-independent effects have been described in recent years. Epidemiological studies indicate that NSAIDs are neuroprotective, although the mechanisms underlying their beneficial effect remain largely unknown. Microglial cells play a major role in brain inflammation and are often viewed as major contributors to the neurodegeneration. Therefore, microglia represent a likely target for NSAIDs within the brain. In the present review, we focused on the direct effects of NSAIDs and selective COX-2 inhibitors on microglial functions and discuss the potential efficacy in controlling brain inflammation.

## 1. Introduction

Inflammation is in the first instance a self-defense reaction that may, under specific circumstances, develop into a chronic state and become a causative factor in the pathogenesis of a broad range of pathologies. In many chronic and disabling diseases, regardless of their primary etiology, inflammation is the main therapeutic target and, often, the best choice to treat the disease and alleviate the symptoms. Like inflammation in peripheral organs, “neuroinflammation” is a two-edged sword, being a self-defensive reaction aimed at eliminating the injurious stimuli and restoring tissue integrity, but also contributing to tissue damage when exceeding critical thresholds. Brain inflammation is tightly controlled and in many but not all cases, it develops as a local process, which is triggered and sustained by resident cells in the absence of overt leukocyte infiltration from blood stream [1]. 

The major players of the brain innate immune response are microglial cells, the macrophages of brain parenchyma. Microglia derive from myeloid precursors that enter the developing central nervous system to become the major population of brain resident macrophages. Under physiological conditions, they show a ramified morphology and lack of cell-surface and cytoplasmic molecules typically associated with other tissue macrophages. In this quiescent or “resting” state microglia are not dormant, but rather they actively “sense” the surrounding parenchyma through their extremely motile processes and protrusions. By doing so, they can react to virtually every environmental change. The reaction or “activation” of microglial cells is typified by cell body enlargement, loss of ramified processes and up-regulation of cell-surface and/or cytoplasmic antigens. In addition, depending on the nature and the intensity of the activating event, microglia can synthesize and release a broad range of different molecules, some of which, e.g., free radicals, inflammatory cytokines, chemokines, lipid mediators, are necessary and sufficient to mount an inflammatory response [2]. 

Although in the past activated microglia have been regarded mainly as detrimental for the surrounding cells, it is now clear that activated microglia play a dynamic, complex and multifaceted role in brain pathology, which needs to be defined within each disease [1,2,3]. In acute brain ischemia, microglia are activated within minutes whereas blood derived macrophages are recruited with a delay of 24–48 hours [4,5,6]. In several ischemia models, expression of inflammatory mediators peaks with the extensive microglial activation, which has been associated to tissue injury [7,8]. A major pathogenic role of the resident microglia as compared to infiltrating macrophages is supported by the observation that depletion of peripheral macrophages by liposome-encapsulated clodronate does not affect infarct size [5]. However, recent studies have documented exacerbation of injury following selective microglial ablation [9], in line with the view of a dynamic role of microglia during the course of disease. In chronic neurodegenerative diseases morphologically activated microglia is a common finding and the increasing interest for the pathogenic role of inflammation/microglial activation in neurodegeneration is exemplified by the growing number of studies on this topic over the last years [10]. Nonetheless, as recently discussed [3], the functional phenotype associated to the morphological activation in chronic neurodegenerative diseases, including Alzheimer’s disease (AD), Parkinson’s disease and amyotrophic lateral sclerosis, is still poorly defined and need further investigations. More recently, the concept of inflammation as a common mechanism of disease has been extended to psychiatric and neurodevelopmental disorders such as depression, schizophrenia and autism spectrum disorders [11,12,13]. 

Non steroidal anti-inflammatory drugs (NSAIDs) are the therapeutic agents of first choice for the treatment of inflammation, pain, and fever. The term NSAID refers to a group of structurally diverse chemical compounds that share the ability to inhibit the activity of the prostaglandin (PG) biosynthetic enzymes, the cyclooxygenase (COX) isoforms 1 and 2. Prostaglandins (PGs) are major pro-inflammatory agents and the suppression of their synthesis at sites of inflammation has been regarded as primarily responsible of the beneficial properties of NSAIDs, although several COX-independent effects have been described in recent years.

## 2. Microglia, PG Synthesis and NSAIDs

Microglia are an important source of PGs ([14] and references therein), and they represent a suitable target for the activity of NSAIDs within the brain. The biosynthesis of PGs begins with the release of arachidonic acid (AA) from membrane phospholipids by either secretory or cytoplasmic phospholipases. Once released, AA is converted via a two-step reaction catalysed by COX enzymes into prostaglandin G2 (PGG2), and subsequently prostaglandin H2 (PGH2). COX-1 and -2 isoforms are unique enzymes as they exhibit two catalytic activities, a bis-oxygenase activity (COX), and a peroxidase activity (POX). The conversion of AA to PGG2 is dependent on a tyrosine-385 radical at the COX site. Formation of a ferryl protoporphyrin IX radical cation from the reducing agent Fe^3+^ at the POX site is essential for conversion of tyrosine-385 to its radical form, and thus to ensure the complete conversion of free AA to PGH2. Finally, PGH2 is converted by specific isomerases and synthases into biologically active primary PGs (PGD2, PGE2, PGF2α, PGI2) and thromboxane A2 (TxA2). In inflammatory conditions, PGE2 is one of the major PGs. Prostaglandin E2 synthases (PGES), the specific isomerase converting PGH2 to PGE2, exists in at least three forms: cytosolic (c) PGES, membrane-associated (m) PGES-1 and mPGES-2. The expressions of COX-2 and mPGES-1 are both induced by inflammatory stimuli such as lipopolysaccharide (LPS), interleukin (IL)-1β, and tumour necrosis factor (TNF)-α, suggesting a functional coupling between COX-2 and mPGES [15,16]. On the other hand, cPGES is constitutive and functionally coupled with COX-1 [17]. The inducible enzyme COX-2 is regarded as the one primarily responsible for PG production in acute and chronic inflammatory conditions. However, a role for the constitutive isoform COX-1 in inflammation has been originally suggested by Langenbach and colleagues [18], who showed that only the dual COX-1/COX-2 inhibitor indomethacin, and not selective COX-2 inhibitors, was able to completely abrogate PGE2 levels at sites of brain inflammation. More recently, genetic ablation or pharmacologic inhibition of COX-1 were shown to reduce inflammation and neurodegeneration in intracerebrally LPS-injected mice [19], whereas COX-1, but not COX-2, inhibitors reverted the behavioral deficits in a mouse model for acute systemic inflammation [20]. Further evidence support a role for COX-1 in neuroinflammation, as extensively reviewed by [21,22]. In the brain, COX-2 is constitutively expressed in excitatory forebrain neurons [23,24], but it rapidly up-regulated mainly in microglia upon activation, both *in vitro* and *in vivo* [25,26,27,28]. In addition to COX-2, activated, but not resting, microglia express mPGES-1 [29,30]. As for peripheral macrophages, the synthesis of PGE2 by microglia exposed to LPS is inhibited by several NSAIDs, with different IC_50_. As shown in Table 1, indomethacin and the COX-2 selective inhibitor NS398 are the most potent inhibitors of PGE2 synthesis in rat microglial cells.

**Table 1:** Effect of NSAIDs on LPS-induced PGE_2_ release by microglial cells.

Drugs	IC50
Indomethacin	1 nM
NS-398	2.5 nM
Flurbiprofen	0.1µM
Piroxicam	0.1µM
Paracetamol	7.6 µM
ASA	10 µM

Rat microglial cells were incubated for 24 h at 37 °C with 10 ng/mL LPS in the presence of increasing concentrations of each drug (0.1 nM–100 µM). None of the drugs tested affected PGE_2_ basal level. IC_50_ values are reported.

In addition to classical NSAIDs, paracetamol (acetaminophen) has been shown to reduce PGE2 synthesis by inhibiting the activity of COX in LPS-stimulated microglial cultures without affecting COX-1 or COX-2 expression, AA release or PGES expression and activity [29]. Paracetamol is an unusual analgesic/antipyretic drug, which acts preferentially by reducing PG production within the central nervous system. Unlike the majority of NSAIDs, paracetamol inhibits COXs only in an environment that is low in peroxides. It acts as reducing co-substrate at the POX site and diminishes the ferryl protoporphyrin IX radical cation availability. This effect can be nullified by the presence of high peroxide tone or by swamping the POX site with substrate such as PGG2 [31]. Peroxide tone and swamping explain lack of peripheral analgesic and anti-inflammatory effect by paracetamol in peripheral organs, as the recruited leukocytes generate high concentrations of cellular peroxides. On the contrary, brain inflammatory response, which often occurs in the absence of leukocytes, provides more favourable conditions to paracetamol activity. Microglial cells show prominent glutathione (GSH) peroxidase and GSH reductase activities and higher GSH content than peripheral macrophages [32,33], suggesting that these cells represent an optimal environment for paracetamol inhibitory activity of the COX-2-dependent PGE2 synthesis. 

In general NSAIDs cross the blood brain barrier (BBB) efficiently [34,35,36], though the effective dose reaching the brain can be different under different neuropathological conditions, depending on BBB integrity. Marked individual variations in response to NSAIDs have been reported in peripheral organs, making it difficult to correlate pharmacokinetic parameters to clinical efficacy [37].

## 3. NSAID-Dependent Activation of Peroxisome Proliferator Activated Receptor-γ in Microglial Cells

The peroxisome proliferator-activated receptor-γ (PPAR-γ) belongs to a large group of nuclear receptors controlling reproduction, metabolism, development and immune response [38]. Among the members of this receptor family, PPAR-γ is the most studied, in part due to its therapeutic potential for treatment of diabetes and related consequences such as metabolic syndrome. PPAR-γ can be activated by both naturally occurring compounds and synthetic agonists. Natural agonists comprise several long chain fatty acids and the cyclopentenone prostaglandin 15-deoxy Δ^12,14^ prostaglandin J_2_. Among the synthetic agonists are the anti-diabetic thiazolidinediones and few NSAIDs, such as indomethacin, ibuprofen and diclofenac. In most cases, however, the NSAID doses required for PPAR-γ agonist activity are in the high micromolar range, thus largely exceeding those required for *in vivo* inhibition of COXs. Among other anti-inflammatory drugs, aspirin and paracetamol lack of any PPAR-γ agonist activity.

Several lines of evidence suggest that PPAR-γ natural and synthetic agonists may control brain inflammation and be of potential therapeutic use in human brain diseases [39]. The beneficial effects of PPAR-γ agonists are mediated by several mechanisms involving anti-inflammatory effects on peripheral macrophages and lymphocytes as well as on microglial cells. In addition, PPAR-γ agonists control functions and survival of other neural cells, including astrocytes, neurons and oligodendrocytes.

Primary microglial cells express PPAR-γ and such basal expression is increased by specific PPAR-γ agonists, while it is reduced in the presence of microglial activators such as LPS and interferon (IFN)-γ [40]. Microglial PPAR-γ is functionally active, being able to bind specific PPRE sequences upon activation by natural and synthetic agonists. Its activation inhibits LPS-stimulated inducible nitric oxide synthase (iNOS) expression and TNF-α production as well as IFN-γ-induced expression of major histocompatibility complex class II antigens, by preventing the activation of the transcription factors STAT-1 and NF-κB [40,41]. 

Some of the neuroprotective effects described for NSAIDs in neurodegenerative diseases or their animal models could therefore be, at least in part, related to PPAR-γ-dependent inhibition of microglial activation [42,43]. In several AD animal models long-term treatment with ibuprofen or indomethacin reduced the extent of microglial activation, with a concomitant reduction in Aβ plaques and inflammatory markers ([42] and following sections). Similarly, in Parkinson’s disease mouse models, indomethacin protected against MPTP induced neurotoxicity and decreased microglial activation [44]. However, several other NSAIDs, with no PPAR-γ agonistic activity are neuroprotective in PD models, as recently reviewed by Esposito *et al.* [45]. 

## 4. Alternative Molecular Targets of NSAIDs

In addition to COXs and PPAR-γ, several alternative molecular targets can account for the broad protective effects of these compounds against a variety of pathological conditions, with or without a prominent flogistic base, including colon cancer, cardiovascular and neurodegenerative diseases. The strongest evidence of COX-independent NSAID activities derives from studies showing anti-inflammatory, analgesic and anti-tumoral effects of the *R*-enantiomers of NSAIDs, which are devoid of COX-inhibitor activity, and from the anti-proliferative effects of NSAIDs on COX-deficient cell lines ([46] and refs. therein). NSAIDs may differentially exert immunomodulatory effects on activated macrophages and lymphocytes at clinically available doses, regulating immune responses such as TNF-α release and nitric oxide (NO) production, cell-cell adhesion, phagocytic uptake, and lymphocyte proliferation [47]. 

In the brain, NSAIDs protective effects against neurodegenerative diseases include, besides COX blockade, inhibition of β-amyloid (1-42) (Aβ42) production and aggregation, inhibition of β and γ-secretase activities, free radical scavenging activity, stimulation of neurotrophin synthesis, and modulation of specific microglial, astrocytic, neuronal, and endothelial cells properties.

As a few examples, sulindac sulfide, indomethacin and meclofenac and both *S*- and *R*-enantiomers of ibuprofen have been reported to lower Aβ42 while increasing Aβ38 fragment production in *in vitro* and *in vivo* models of AD, by COX-independent mechanisms involving microglia as well as neighbouring cells (for extensive reviews see [42,48]). NSAIDs can act at the neuronal level by directly targeting presenilin 1, the catalytic component of the γ-secretase complex that cleaves among other membrane proteins the amyloid precursor protein (APP), or modulating the secretase BACE1 via PPAR-γ dependent mechanisms. In turn, the lowering of Aβ products, which are regarded as pro-inflammatory activators of microglia and monocytes, could dampen microglial activation, a phenomenon described in the majority of the studies of long-term administration of indomethacin or ibuprofen in AD models. 

In addition, NSAID-dependent modulation of microglial phagocytosis, could contribute to plaque and debris removal and to the lower plaque burden and gliosis observed following long-term NSAID treatment [49,50]. In an *in vitro* study evaluating the effects on microglial activation of opsonization of Abeta deposits with anti-Aβ IgG, as a strategy to enhance microglial clearance of Aβ deposits, indomethacin had negligible effects on microglial migration and phagocytosis of Aβ, but it did significantly inhibit microglial secretion of IL-6 following opsonization [51]. In other few studies, indomethacin, naproxen and the selective COX-2 inhibitors, SC-236, SC-245 and SC-791, reduced phagocytic activity of microglia or resident rat peritoneal macrophages [52,53]. Prolonged exposure to celecoxib, a COX-2 selective inhibitor, has recently been shown to disrupt microglial rafts, induce aberrant receptor recruitment to rafts and impair receptor-mediated phagocytosis [54]. These conflicting results may be attributed to the experimental models adopted and to the specific compounds and concentrations used. In any case, they reflect the incomplete understanding of NSAID effects on microglia/macrophage phagocytosis.

To reconcile the apparent conflicting results of epidemiological, experimental and clinical studies on the efficacy of NSAIDs in AD, it has been proposed that the chronic use of NSAIDs may be neuroprotective only as a prevention strategy whereas, in later phases of AD pathology, NSAIDs use may even turn detrimental, also because of their inhibiting activity on activated microglia [43]. Indeed, as mentioned earlier, microglial activation is a complex and multifaceted process that encompasses a plethora of functional states, bearing also potentially protective and regenerative functions. The selective inhibition of the cytotoxic microglial functions in the opportune time window, while preserving the protective phenotypes could be more productive, for the accomplishment of an efficient repairing response to damage, than inhibiting the process of microglial activation as a whole. 

The need of a selective modulation of microglial activation is suggested also by studies analysing the role of microglial activation in the regulation of endogenous neurogenesis and the potential use of NSAIDs to enhance regenerative processes. From these studies, it has emerged that whether acute inflammation impairs both basal and insult-induced neurogenesis, chronically activated microglia can acquire a protective pro-neurogenic phenotype that can promote the regenerative process (for recent reviews see [55,56]).

A further note of caution in the use of COX-2 selective inhibitors (coxibs) and nonselective NSAIDs as neuroprotective agents rises from findings about their ability to up-regulate vascular NADPH oxidases and superoxide and nitric oxide (NO) production, thus inducing oxidative stress [57]. Microglial NADPH oxidase has been involved as a primary source of ROS and oxidative damage found in both AD brains and mouse models of neurodegenerative diseases [58], suggesting that long term use of these compounds could worsen tissue damage. 

NSAIDs, by analogy to their antiproliferative activity on cancer cells, might inhibit microglial proliferation by modulating the cell cycle progression and apoptosis [59], thus representing a further mechanism by which these compounds can affect the innate immune response in the brain. In view of the above considerations on the role of microglia in brain inflammation, it remains to be established if this effect can be considered as always beneficial.

Additional mechanisms by which NSAIDs can affect microglial activation process include their ability of modulate the activity and/or expression of crucial signaling molecules. A subset of NSAIDs (sodium salicylate, ASA, flurbiprofen, and the selective COX-2 inhibitor rofecoxib), are able to target NF-κB [60,61,62], a transcription factor involved in the control of cell growth, survival, plasticity, memory formation, cognition and behavior, and modulation of β-secretase (BACE1) expression [63,64]. In microglia NF-κB is a key signaling molecule, which regulates the transcription of pro-inflammatory genes, immunoreceptors, metalloproteinases and cell adhesion molecules [65]. The selective expression of specific genes among the over 150 NF-κB target genes so far recognized, depends on the concomitant regulation of other transcription factors, such as the activator protein (AP)-1, which has also emerged as a target for some NSAIDs (sodium salicylate, ASA, flurbiprofen), but not of the COX-2 selective inhibitor refecoxib [46,62,66]. Other COX-2 selective inhibitors, such as celecoxib, have been shown to activate NF-κB, at least at high doses in rat renal mesangial cells [67], suggesting caution in the generalization of the protective potential of these NSAIDs in all pathological settings.

Another target of some NSAIDs (ASA, aspirin, ibuprofen, NS-398) is the mitogen activated protein kinase (MAPK) p38 [68], which plays a crucial role in regulating the microglial synthesis of pro-inflammatory molecules such as TNF-α and NO [69]. Its activation leads to the phosphorylation of a multitude of downstream kinases and transcription factors, regulates mRNA stability of proinflammatory cytokines such as TNF-α, and affects chromatin accessibility to transcription factors such as NF-κB. Therefore, altered p38 activation by NSAIDs may provoke a profound reprogramming of the signaling network related to this kinase in microglia. A protective effect of rofecoxib, presumably mediated through the inhibition of p38MAPK phosphorylation, has been reported in a model of excitotoxic degeneration *in vivo* [70].

## 5. Nitric Oxide- and Hydrogen Sulfide-Releasing NSAIDs

Beside their potent anti-inflammatory activities, NSAIDs are associated with significant adverse effects on gastrointestinal tract and kidneys, which pose critical limits to their clinical use in chronic conditions. In the last decades there has been an increasing effort to develop “safe” NSAIDs. 

One strategy has led to the development of COX-2 selective inhibitors or coxibs, aiming at sparing the physiological activity of COX-1, which is expressed constitutively in several tissues including gastric epithelium. Coxibs have been rapidly introduced in the clinical practice on the ground of their reduced risk of gastrointestinal bleeding compared to non-selective COX inhibitors such as classical NSAIDs. However, they have later shown significant cardiovascular adverse effects, eventually leading to the withdrawal from the market of one drug of this class (rofecoxib) and the introduction of some limitations to their clinical use [43,71]. 

Another strategy has been to modify classical NSAIDs by grafting NO-donating moiety onto the NSAID scaffold using different chemical spacers [72,73]. The release of NO from these compounds occurs through slow kinetics, mimicking the physiological levels of NO produced by constitutive NOS, and thus protecting the gastrointestinal mucosa [74,75]. This modification strongly reduces their untoward side effects without altering the anti-inflammatory effectiveness.

We reported that one of these NO-donating NSAID (NO-flurbiprofen) is as potent as flurbiprofen in preventing PGE_2_ synthesis in LPS-activated microglial cultures [76]. Moreover, NO-flurbiprofen enhanced the expression of iNOS in LPS-challenged microglia; this effect was independent from flurbiprofen activity and most likely attributable to the NO released from the drug, as NO-donors lead to similar results. In LPS-activated microglial cultures, at variance with previous observations on peripheral macrophages [77], flurbiprofen and NO-flurbiprofen caused a similar moderate reduction of LPS-induced IL-1β release, indicating that NO-NSAIDs may differently affect peripheral and brain macrophages [76].

In a follow-up of these studies, Bernardo *et al.* [78,79] demonstrated that two NO-releasing derivative of flurbiprofen, HCT1026 and NCX 2216, were able to activate PPAR-γ and induce its specific binding to PPRE sequence. As expected, the native compound flurbiprofen was unable to activate PPAR-γ, suggesting that structural modifications due to the grafting of the NO-donating moiety are responsible for the PPAR-γ agonistic activity of HCT1026 and NCX 2216. The two drugs activated PPAR-γ with different kinetics and their activities were abolished by the presence of the specific PPAR-γ antagonist GW9662. Interestingly, NCX 2216, which differs from HCT 1026 for the presence of a NO-donating moiety having antioxidant activity, after an initial activation, induced PPAR-γ nitration and inactivation. These effects on PPAR-γ were paralleled by a transient reduction of TNF-α and NO production and a protracted inhibition of IL-1β and PGE_2_ synthesis, suggesting a dynamic regulation of the functional state of activated microglia by NCX 2216. Long term treatment with NCX 2216 could therefore, after the initial activation of PPAR-γ, result in a partial escape of microglia from the negative control of PPAR-γ. These observations help explaining why among the few NSAIDs with Aβ-lowering activity in AD animal models, only in the case of protracted administration of NCX 2216 the reduction of cerebral amyloid load is accompanied by a sustained microglial activation in the peri-plaque area [80,81].

The same approach of grafting a protective moiety onto a classical NSAID has been used to develop a further class of compounds called S-NSAIDs or hydrogen sulfide (H_2_S)-releasing NSAIDs [82]. These compounds are metabolized by carboxylesterases in the body to slowly generate H_2_S, so that high-dose-induced side effects H_2_S can be prevented. Hydrogen sulfide is a gas that can be formed by the action of two enzymes, cystathionine γ-lyase and cystathionine β-synthase, both involved in the metabolism of cysteine. In analogy with nitric oxide and carbon monoxide, H_2_S is regarded as a “gas-transmitter” with signalling functions in several biological systems [83].

Hydrogen sulfide is produced endogenously and has multiple functions especially in the brain, where is protective against oxidative stress in neurons [84] and reduces the inflammatory response, likely by inhibition of p38 MAP kinase as shown in LPS-activated microglial cultures [85]. 

Lee and colleagues [86] have found that the H_2_S-releasing molecules ADT-OH, *S*-diclofenac, and *S*-aspirin protected the neuronal cell line SH-S5Y5 against LPS or INF-γ activated microglial toxicity. The pre-treatment of microglial cultures with *S*-NSAIDs reduced the release of the pro-inflammatory mediators TNF-α, IL-6, and NO. The *S*-NSAIDs showed enhanced activities as compared to NSAID or sulfur containing parent compounds. The activities of NSAID molecule and H_2_S-donating moiety were additive, suggesting that two independent anti-inflammatory mechanisms were simultaneously activated. Hydrogen sulfide is a reducing agent that can increase intracellular GSH levels, a major intracellular antioxidant [84]. Moreover, previous studies demonstrated that endogenous H_2_S may scavenge peroxynitrite and reduce its toxicity [87]. The mechanisms by which H_2_S moiety exerts anti-inflammatory activities and limit microglial activation are, however, not yet fully elucidated.

## 6. Natural Anti-Inflammatory Drugs

Several natural anti-inflammatory drugs have been identified in plant extracts used in traditional medicine for the relief of pain, fever and inflammation. In the past few years, the mechanisms of some of these natural compounds have been partly elucidated, and they are now being reconsidered for treatment of chronic inflammatory and neurodegenerative diseases. In most cases these drugs work by inhibiting the transcription of COX-2 rather than its activity. In addition they prevent the expression of several pro-inflammatory genes.

To mention a few examples, torilin, obtained from *Ulmus davidiana* japonica, inhibited LPS-induced iNOS, COX-2 and IL-1β in the microglial cell line BV2, via down-regulation of ERK1/2, p38 MAPK, NF-κB and CREB [88]; 4-methoxyhonokiol, an active anti-inflammatory constituent of the bark of *Magnolia obovata*, significantly inhibited LPS-induced expression of iNOS and COX-2 in RAW 264.7 macrophages, acting on JNK and p38 MAPK signal pathways and NF-κB activation [89]. Finally, studies performed in primary microglial cultures showed that macelignan, a compound extracted from *Myristica fragrans*, inhibits iNOS expression at the transcriptional level [90] and the production of TNF-α and IL-6. As for 4-methoxyhonokiol, macelignan suppressed both MAPK phosphorylation and NF-κB activation in LPS-stimulated BV-2 microglial cells [91].

## 4. Conclusions

**Figure 1:**
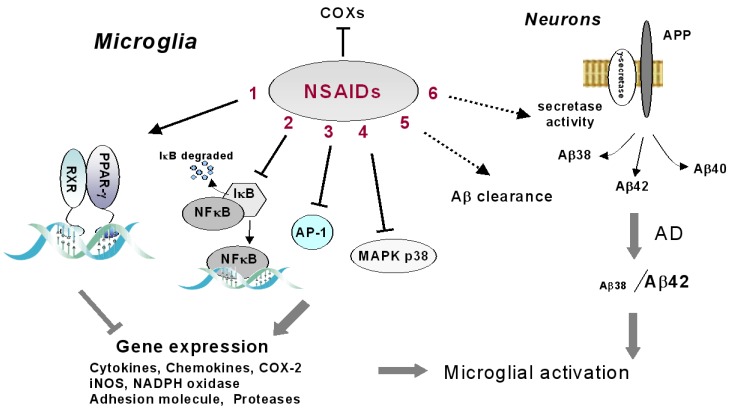
Schematic representation of direct and indirect effects of NSAIDs on microglial functions. Besides inhibiting COX activity, some NSAIDs can repress the expression of genes typically associated with microglial activation by interfering with NF-kB, AP-1 and MAPK p38 signaling, and activating PPAR-γ. NSAIDs can also indirectly limit microglial activation, by interfering with secretase activity in neurons and reducing Aβ load. The effects of NSAIDs on microglial Aβ clearance are still controversial (dotted line). (1) ibuprofen, indomethacin, diclofenac; (2) sodium salicylate, ASA, flurbiprofen, rofecoxib; (3) sodium salicylate, ASA, flurbiprofen; (4) ibuprofen, ASA, aspirin, NS-398; (5) ibuprofen, indomethacin, naproxen and the selective COX-2 inhibitors, SC-236, SC-245 and SC-791, celecoxib; (6) ibuprofen, indomethacin and meclofenac, sulindac sulfide.

The recognition of inflammation as a common mechanism of disease in neurological and neuropsychiatric diseases has brought to an increasing interest of the neuroscience community on anti-inflammatory drugs. NSAIDs, a group of heterogeneous molecules that accounts for a large share of the drug market, have been proved neuroprotective in a number of experimental settings, however their efficacy in human brain diseases remains controversial. Originally described as COX inhibitors, NSAIDs might affect a multitude of signaling pathways and cellular mechanisms, which are not yet fully understood. In this review article, we have tried to illustrate the possible impact of these drugs on brain inflammation through their actions on microglial cells (Figure 1). Many important issues remain unexplored. For example, the consequences of their amphiphilic nature, which allows NSAID interaction with lipid membranes, on the modulation of membrane biomechanical properties and cell signaling events [92], or other interesting molecular mechanisms that have been described as relevant for the chemopreventive effects of several NSAIDs. Among these, the induction of a gene belonging to the TGF-β superfamily, with proapoptotic properties, the nonsteroidal anti-inflammatory drug-activated gene-1 (NAG-1) [93], could be relevant in the modulation of microglial activation and deserve to be evaluated in future studies. A deeper knowledge of the diversity of NSAID actions as well as of the multifaceted role of inflammation and microglial activation in brain diseases will help in filling the gap between experimental and clinical results and in translating our knowledge into successful brain disease treatment.
